# Editorial: “Global Challenges in Radiation Oncology”

**DOI:** 10.3389/fonc.2015.00103

**Published:** 2015-05-15

**Authors:** Daniel Grant Petereit, C. Norman Coleman

**Affiliations:** ^1^Walking Forward Program, Rapid City Regional Cancer Center, Rapid City, SD, USA; ^2^International Cancer Expert Corps, New York, NY, USA; ^3^Radiation Research Program, Division of Cancer Treatment and Diagnosis, National Cancer Institute, Rockville, MD, USA

**Keywords:** editorial, radiation oncology, global cancer disparities, LMICs

## Introduction

In the United States, much of the research is focused on developing new and expensive technologies and drugs that are of great scientific and clinical interest, but usually providing incremental therapeutic benefit. In contrast, in resource-limited countries, basic oncology care is frequently lacking. In addition, the outcomes from various chemo–radiotherapy combinations for a number of malignancies are unknown, as these populations have not been adequately investigated. For oncologists in these countries who have marginal to barely adequate resources, accrual to clinical trials is virtually non-existent because of the complexities of social and economic issues facing their population, competing co-morbidities and lack of access. As a result, there is a tremendous disparity in outcomes for these populations, as compared to those in developed countries.

At first, it may appear odd that radiation oncologists, often associated with high-cost technology, would have leading role in global cancer disparities. However, radiation is a critical treatment modality for the majority of cancers whether the intent is curative or palliative. In fact, a single dose of palliative radiotherapy is more cost effective than a prolonged course of narcotics (1). In addition, for many solid malignancies observed in low to middle income countries (LMICs), such as breast, cervical, head and neck (H&N), upper GI, central nervous system (CNS), and lung cancers, radiation will achieve very effective palliation, and sometimes cure, even when concurrent chemotherapy cannot be given or when oncologic surgeons are unavailable. In addition, radiation oncology centers are often the hub of technologies, such as telemedicine, which can facilitate collaboration with other cancer centers worldwide.

The authors are privileged to be guest editors for this Frontiers Research Topic highlighting the issues addressing global cancer disparities. The authors have asked a number of oncologists from different parts of the world to report their experience and thank them for their time and work over the last year.

Topics covered include systematic review of radiation resources in low and middle income countries, planning national radiotherapy services, human resources for cancer control in Uttar Pradesh, India, locally advanced breast and cervical cancer (India, Africa), patient navigation, the challenges of performing clinical trials in South Africa, the cervical cancer research network (CCRN), the US Cancer Disparities Research Partnership (CDRP), training radiation oncologists in underserved parts of the world, and building sustainable partnerships through the newly formed International Cancer Export Corps (ICEC). The authors discuss “lessons learned” from their populations, practical suggestions to address these disparities, and how we as a global oncology community can address and potentially mitigate these global challenges.

According to the World Bank classification, 139 countries are considered LMICs as their gross national income (GNI) per capita is ≤USD 12,615 (2). The World Health Organization (WHO) report in 2010 and the United Nations declaration in 2012 chronicled the growing burden of non-communicable diseases (NCD) in the developing world (3, 4). In the past decade, the global incidence of cancer has increased by 20%, mostly because of cases in LMIC (5). By 2020, up to 70% of the 20 million new cancer cases are expected to occur in these countries (6). Furthermore, these countries are not prepared to address this cancer epidemic, and consequently, cancer survival rates are less than one-third of those for site specific cancer types in high-income countries. It is imperative that they develop and sustain the infrastructure needed to prevent, diagnose, and treat this cancer “tsunami” (7). Case burdens are also increasing in rural underserved areas in resource-rich countries with the native/aboriginal populations often having similar access and care issues as LMICs, as the Northern Plains American Indians (AIs) have the highest cancer mortality rate in the United States (8–10).

Cervical cancer is of global interest as almost 85% of the worldwide 530,000 cases in 2012 were diagnosed in developing countries. This is amenable to detection by screening and potentially preventable with vaccination (11, 12). Furthermore, even patients with advanced stages of cervical cancer are still curable if appropriate radiation doses can be given with a combination of external beam radiation and brachytherapy (13). The social and economic impact is substantial as cervical cancer disproportionately affects young women (14–16).

The International Atomic Energy Agency (IAEA) recommends a teletherapy unit, a radiation oncologist, a medical physicist, and two radiotherapists (RTTs) per 250,000 people (17, 18). The inadequacy of radiation oncology services for LMICs is reported by Grover et al. in a systematic review of five international databases. A world map of current teletherapy units from the IAEA is depicted in Figure 1 from the Rosenblatt article (18). In many parts of Africa, there is only one teletherapy unit per 10 million people! The inadequacy of radiation therapy infrastructure from the IAEA–DIRAC database was recently reported by Datta et al. (19). They estimated by 2020, 84 LMICs will need 9,169 teletherapy units, 12,149 radiation oncologists, 9,915 medical physicists, and 29,140 radiation therapy technologists. It is estimated that Africa is functioning at 25% of its potential for treating cervical cancer (20). These projected needs are simply staggering and cannot be allowed to stand.

Determining the human resources needed to treat cancer is a critical first step as it is important to guide investment and progress (21). Daphtary et al. (22) describe a unique methodology for estimating these resources needed in the state of Uttar Pradesh, India, with a population of 200 million. Using the publicly available sources of GLOBOCAN^1^ and city population^2^, they explain an enormous shortage of human and other resources for cancer control (12, 23). As the data was generated from 2008, the dilemma is expected to be even more dire as the cancer cases in India is projected to increase by 30% over the next 10 years. This case study of Uttar Pradesh may serve as a road map for other interested stakeholders and policy makers in a variety of LMICs.

Rosenblatt indicates that there should be a systematic and ­comprehensive process of long-term planning of radiotherapy services at the national level, taking into account the regulatory infrastructure for radiation protection, planning of centers, equipment, staff, education programs, quality assurance, and sustainability aspects. He adds that “realistic budgetary and cost considerations must also be a part of the project proposal or business plan”.

In the second article by Grover and colleagues, the need to train global oncologists from the perspective of a US resident is presented. There is an interest and potential need for US residents to have global training experience, and a concomitant urgent need for LMIC countries to develop oncology training, infrastructure, and services, possibly in collaboration with US residents. Although limited but growing, there are international options for US residents including The Paul Famer Global Surgery Fellowship, international pediatric oncology twinning programs, travel grants through the American Society of Radiation Oncology (ASTRO), and the Global Health Scholars Program through ASTRO-Association of Residents in Radiation Oncology.

Although this “tsunami” of cancer in LMICs is overwhelming and seemingly hopeless, a recent delegation of radiation oncologists, residents, and medical physicists embarked on a mission to the city of Dakar, Senegal West Africa, to implement the first high-dose-rate (HDR) remote afterloader, as this country of 13 million people only had a single Cobalt teletherapy unit with no brachytherapy services. By partnering with Radiating Hope, a non-profit organization whose mission is to update and provide radiation equipment to developing countries and founded by Dr. Brandon Fisher, the first cervical cancer patients were treated with curative intent. This “beacon” of hope may serve as a model and inspiration for other LMICs (24, 25) but is only 1/5000th or so of the need.

Conducting clinical trials for common disease sites in LMICs is of critical importance as the data generated from other countries may not be applicable for these populations. Dr. Roy Lakier, an oncologist from South Africa, kindly shared his data that chronicled the tribulations of an IAEA sponsored phase III trial investigating radiation alone versus chemo-radiation for HIV positive cervical cancer patients. Even with minimal resources to conduct research, they successfully enrolled 81 patients. No clinically relevant conclusions could be drawn because of “relatively” small numbers and incomplete follow-up. Twenty percent of patients were lost to follow-up and 6% died during the first 6 months reflecting advanced stages of disease, impaired nutritional status, and significant medical co-morbidities. Their experience detailed several problematic areas including inadequate radiation therapy equipment, delays in obtaining pathology and imaging promptly, unavailability of chemotherapy drugs, transportation, social and medical co-morbidities, and non-supportive hospital policies with the extra research expenses incurred. Lakier and his co-workers are to be commended for conducting this phase III trial in a very resource-limited environment.

As evident by Lakier, access to cancer clinical trials is scare in LMICs with limited to unavailable research support and infrastructure. The Cervix Cancer Research Network (CCRN) was developed as a potential solution whose overall goal is to promote cervical cancer research and improve access to novel therapies. Of course, basic radiation services are a pre-requisite before novel therapies are considered. The CCRN is a subsidiary of the Gynecologic Cancer Intergroup (GCIG), and was developed under the vision of of Dr. Henry Kitchener from the University of Manchester. As described by Suneja, 17 CCRN site visits have been performed with four multinational clinical trials opened that were deemed suitable. They suggest the use of cell phone technology to increase patient compliance which was problematic in Lakier’s experience. We recently implemented a mobile health technology (mHealth) randomized trial using customized text messaging, counseling, and nicotine replacement to address the high smoking rates among the Northern Plains American Indians (26). In this resource-limited population, recruitment and compliance to this trial has been high. Therefore, the use of mHealth technology for LMIC populations for treatment compliance, follow-up, and clinical trials may be a potential solution.

The disparity of breast cancer in LMIC is evident as it occurs in younger women who present with a higher incidence of locally advanced breast cancer (LABC) when compared with women from developed countries as discussed by Balogun and Formenti. They make the case that “financial resources are likely better invested in public awareness campaigns and training community health workers to educate the public and perform clinical breast exams (CBE) rather than screening mammography” (27–29). Basic chemotherapeutic agents such as paclitaxel, doxorubicin, cyclophosphamide, and tamoxifen, rather than expensive targeted therapy such as herceptin, are recommended for systemic therapy. The dire need for adjuvant external beam radiation is discussed in the context of hypofractionation and concurrent with chemotherapy in order to maximize resources.

To increase access of underserved/health disparate communities to NCI clinical trials, the Radiation Research Program (RRP) piloted a unique model – the Cancer Disparities Research Partnership (CDRP) program. CDRP targeted community hospitals with a ­limited past NCI funding history and provided funding to establish the infrastructure for their clinical research program. Wong summarizes the results from the initial six CDRP institutions. Key findings from these community-based hospitals include enrolling ~2,300 patients to clinical trials with ~5,100 patients receiving patient navigation (PN) once the infrastructure was established. Another finding is the need for the cooperative groups to develop clinical trials for locally advanced cancers observed in these disparate populations.

American Indians experience tremendous cancer disparities with the highest 5 year mortality rates when compared with other US races (10). PN is a method to mitigate this disparity as presented by Burhansstipanov and co-workers. According to the Affordable Care Act where a navigator is an “insurance broker”, the true model of patient navigation, as created by Freeman, is one who helps patients overcome barriers to accessing and using a specific health care system (30). Burhansstipanov describes a unique model of PN where navigators are AI and part of the community who navigate in a culturally appropriate fashion. In South Dakota, the authors implemented a similar model of PN for the AI community (Walking Forward) where they were able to document improved satisfaction with the health care system and improved treatment compliance for AIs undergoing radiation (8, 31).

## International Cancer Expert Corps (ICEC): Building a Sustainable Global Network

Likely because of the magnitude of the problem, when global cancer disparities are discussed, often only the problem is presented, rather than solutions and a logical plan to address these complex economic, social, political, and healthcare inequality issues. Signaling a transformational change to respond both to the global need and to create a sustainable altruistic component to healthcare careers, Coleman and colleagues detail the newly formed ICEC whose goal is to reduce the mortality and improve the quality of life for cancer patients in LMIC. They outline key steps in this process including structured support for dedicated faculty attempting to establish a formal career path, with metrics for human service.

The goal for an ICEC Center, within the LMIC, or geographic-access limited setting within resource-rich countries, as often encountered with indigenous populations, is to develop and retain a high-quality sustainable workforce who can provide best possible cancer care for their setting, conduct research, and become a regional center of excellence from which to help other ICEC Centers develop. An international mentoring network of cancer professionals, including many of the contributors to this issue of Frontiers, will work with local and regional in-country groups on projects to develop and sustain expertise and local solutions for better cancer care, as detailed in Figure 1 of the Coleman article (32). The vision is a world in which everyone has access to cost-effective interventions to prevent and treat cancer and its symptoms in ways that are consistent with best possible practices for the local circumstances.

Partnering with and enhancing ongoing global health programs and “twinning” between programs in resource-rich and health disparity communities is an essential tenet of ICEC to help create a critical mass of sustainable expertise, which is difficult to obtain from the independent well-intended smaller programs (i.e., the current model). In essence, ICEC is aiming to create a “public health oncology” road map to “tap into” a global panel of experts to mentor physicians, nurses, scientists, epidemiologists, and other health care and health policy workers from LMICs (33). Global expertise will include academicians, private practitioners, and senior mentors who along with their institution are willing to commit time so that person-to-person relationships will enhance investment in and quality of cancer care where there is a need that must be met by the global community.

Although cancer at the cellular and molecular level is a complex disease that requires multiple interventions for a successful outcome, so too is cancer at the global level as multiple partners are required to address multiple barriers to mitigate these ongoing global cancer disparities. The contributors and their colleagues and partners in this issue of Frontiers are agents of change, addressing a problem that some might consider “too hard”, or “too expensive” … and they are demonstrating that with dedication, support, and commitment, change will occur. Two quotes come to mind from those who have changed the world. Margaret Meade noted: “Never believe that a few caring people can’t change the world. For, indeed, that’s all who ever have”. The authors believe there are a growing number of dedicated and passionate individuals who will transform global oncology sometime in the not too distant future. The authors in the cancer community will smile when they think of the remark by Nelson Mandella, “It always looks hard until it is done!”

## Conflict of Interest Statement

The authors declare that the research was conducted in the absence of any commercial or financial relationships that could be construed as a potential conflict of interest.

## Footnotes

^1^http://globocan.iarc.fr/

^2^http://www.citypopulation.de/
